# RNA-binding protein RBM47 in health and disease: molecular mechanisms, preclinical evidence, and translational challenges

**DOI:** 10.3389/fimmu.2026.1863629

**Published:** 2026-07-09

**Authors:** Shihua Huang, Qingsong Wang, Junhong Shen, Xianmin Wang, Tongyong Luo, Jun Yin

**Affiliations:** 1 Department of Neurology, West China Hospital Sichuan University Jintang Hospital, Jintang First People’s Hospital, Chengdu, Sichuan, China; 2 Department of Pediatrics, West China Hospital Sichuan University Jintang Hospital, Jintang First People’s Hospital, Chengdu, Sichuan, China; 3 Department of Endocrinology, West China Hospital Sichuan University Jintang Hospital, Jintang First People’s Hospital, Chengdu, Sichuan, China; 4 Pediatric Cardiology Center, Sichuan Provincial Women's and Children's Hospital / The Affiliated Women's and Children's Hospital of Chengdu Medical College, Chengdu, Sichuan, China; 5 Department of Ultrasound, West China Hospital Sichuan University Jintang Hospital. Jintang First People’s Hospital, Chengdu, Sichuan, China

**Keywords:** epithelial-mesenchymal transition, post-transcriptional regulation, prognostic biomarker, RBM47, RNA-binding proteins, therapeutic target, tumor microenvironment

## Abstract

RNA-binding motif protein 47 (RBM47) is an evolutionarily conserved multifunctional RNA-binding protein. It mediates post-transcriptional regulation through C-to-U RNA editing, alternative splicing, and messenger RNA (mRNA) stability control. Preclinical evidence indicates that RBM47 exhibits context-dependent, dual effects in human disease. In breast cancer (BC), colorectal cancer (CRC), renal cell carcinoma (RCC), papillary thyroid carcinoma (PTC), and non-small cell lung cancer (NSCLC), experimental studies suggest that RBM47 exerts tumor-suppressive activity through the inhibition of the Wnt/β-catenin, phosphoinositide 3-kinase (PI3K)-AKT, and nuclear factor erythroid 2-related factor 2 (Nrf2) pathways. Conversely, in glioma and pancreatic cancer (PC), cell and animal models indicate that RBM47 exerts oncogenic activity by promoting M2 macrophage polarization and immune evasion. Furthermore, aberrant RBM47 expression has been implicated in postoperative cognitive dysfunction (POCD), inflammatory bowel disease (IBD), and antiviral innate immune regulation in preclinical models. This review summarizes the molecular characteristics and pathological mechanisms of RBM47, discusses the preclinical rationale for its potential utility as a biomarker and therapeutic node, and critically analyzes current research limitations, conflicting evidence, and translational bottlenecks. All biomarker and therapeutic applications discussed remain at the preclinical or retrospective-correlative stage; no RBM47-targeted intervention has entered clinical trials.

## 1 Biological characteristics of RBM47

### 1.1 Molecular features

RNA-binding motif protein 47 (RBM47) is a highly conserved multifunctional RNA-binding protein in vertebrates. It belongs to the RNA recognition motif (RRM) protein family and is classified within the heterogeneous nuclear ribonucleoprotein (hnRNP)-R-Q superfamily (1). The human RBM47 gene maps to the reverse strand of chromosome 4p14 and spans approximately 206.59 kb; the mouse ortholog is located on chromosome 4 (2). The gene transcribes a mature full-length messenger RNA (mRNA) containing 4 coding exons, which is translated into a protein of 593 amino acids with a molecular weight of approximately 64–70 kDa (1, 3).

RBM47 contains three tandemly arranged classical RRM domains (RRM1, RRM2, RRM3) and a C-terminal alanine-rich domain (4). Each classical RRM domain folds into a β1-α1-β2-β3-α2-β4 topology of approximately 90 amino acids. Two central β-strands carry the highly conserved ribonucleoprotein motifs RNP1 and RNP2, which provide the structural basis for RNA recognition. Cross-species alignment shows that the RRM regions of RBM47 are highly conserved among humans, mice, rats, chickens, *Xenopus*, and zebrafish. Zebrafish RBM47 shares 81.5% sequence identity with the human protein, and RRM region similarity reaches 90% (1). Phylogenetic analysis indicates that the RBM47 gene originated during vertebrate speciation and has remained highly conserved (1).

RBM47 exhibits a bipartite nucleocytoplasmic distribution. Immunoblot analysis shows that RBM47 protein is present in both cytoplasmic and nuclear fractions, with enrichment in the nuclear fraction (nuclear-to-cytoplasmic ratio approximately 2:1). This pattern suggests independent roles in nuclear RNA metabolism and cytoplasmic RNA processing (5). The localization has been validated in human induced pluripotent stem cells, human skin fibroblasts, and intestinal epithelial cells. In addition, RBM47 interacts with the mitochondrial antiviral-signaling protein (MAVS) through its N-terminal RRM domain (6).

**Figure 1:**
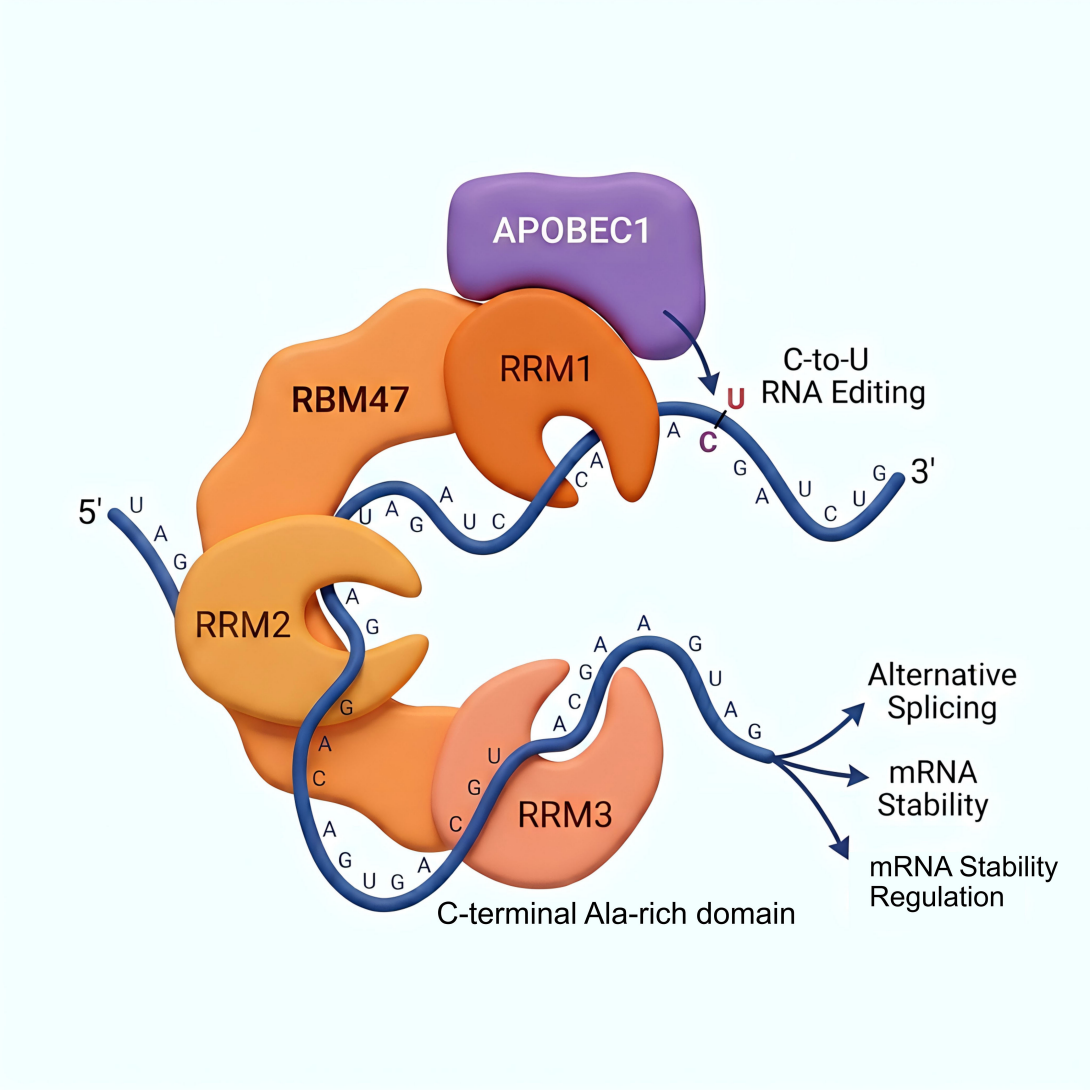
Molecular structure and post-transcriptional regulatory mechanisms of RBM47. RBM47 utilizes its three tandem RNA recognition motifs (RRM1, RRM2, and RRM3) to specifically bind to target mRNAs. Functioning as a critical cofactor, RBM47 assembles with the APOBEC1 complex to drive C-to-U RNA editing. Furthermore, RBM47 orchestrates a highly coordinated post-transcriptional network—including the modulation of alternative splicing and the predominant enhancement of mRNA stability—to precisely govern downstream gene expression profiles. In specific pathological contexts (e.g., PD-L1 in NSCLC), RBM47 can also promote selective mRNA degradation.

Based on analysis of the Genotype-Tissue Expression (GTEx) database, human RBM47 transcripts are highly expressed in the thyroid, adrenal gland, salivary gland, esophageal mucosa, kidney, stomach, small intestine, lung, spleen, liver, and pancreas, whereas expression is low in brain regions, skeletal muscle, and cardiac tissue (1). Single-cell transcriptome sequencing data show that RBM47 is enriched primarily in microglia in the central nervous system (7) and in differentiated enterocytes in intestinal tissue, with lower expression in intestinal stem cells and Paneth cells (8). In adult mice, RBM47 protein is expressed mainly in endoderm-derived organs (small intestine, pancreas, liver, and lung) and is not detected in the brain, heart, or skeletal muscle (4). The Genome-Wide Association Study (GWAS) catalog has recorded 11 single nucleotide polymorphisms (SNPs) in the RBM47 gene. The missense variant rs35529250 (Gly538Arg) is associated with hypertension (1) (**Figure 1**).

### 1.2 Expression regulatory mechanisms

The expression and functional activity of RBM47 are under multi-level control, including transcription factors, epigenetic modifications, post-transcriptional regulation, and inducible expression.

At the transcriptional level, multiple transcription factors participate in positive regulation of RBM47. RUNX1 (runt-related transcription factor 1) binds directly to the promoter region of the mouse *Rbm47* gene and enhances promoter activity; dual-luciferase reporter assays confirmed this direct binding. Under anesthesia and surgical stress, RUNX1 expression increases in the hippocampus and drives RBM47 upregulation, forming a RUNX1-RBM47 axis (9). FOXO3 (forkhead box O3) binds directly to the RBM47 promoter and activates its transcription; in PTC, this regulation participates in the RBM47-SNHG5-USP21-FOXO3 positive feedback loop (10). FOXA1 (forkhead box A1), a key transcription factor for endoderm development, binds multiple sites in the RBM47 promoter and induces RBM47 transcription; chromatin immunoprecipitation quantitative polymerase chain reaction (ChIP-qPCR) confirmed FOXA1 enrichment at predicted binding sites (11). The nuclear factor kappa B (NF-κB) pathway also participates in positive regulation: the NF-κB p65 subunit binds directly to the RBM47 gene promoter, and in glioma, a positive feedback loop forms between RBM47 and NF-κB (12). Negative regulation involves the epithelial-mesenchymal transition (EMT)-associated transcription factors STAT3 (signal transducer and activator of transcription 3) and SNAIL, which bind the RBM47 promoter and suppress its expression (13).

Epigenetic modification is another important layer of RBM47 regulation. DNA methylation has a key role in CRC: in mesenchymal-like cell lines (e.g., SW480, SW620), the RBM47 promoter is fully methylated and the gene is silenced, whereas in epithelial-like cell lines (e.g., DLD1, HCT15) the promoter is unmethylated. The DNA methyltransferase inhibitor 5-azacytidine (5-Aza) partially restores RBM47 expression (11, 13). In RCC, RBM47 expression is regulated by CREB-binding protein (CBP)/E1A binding protein p300 (P300)-mediated histone H3 lysine 27 acetylation (H3K27ac); the RBM47 promoter is enriched with H3K27ac, and this modification is markedly reduced in RCC tissue compared with normal tissue (2).

Post-transcriptional regulation is mediated mainly by microRNA (miRNA). miR-181c-5p and miR-181d-5p target the 3′-untranslated region (3′-UTR) of RBM47 mRNA directly and inhibit its expression; dual-luciferase reporter assays confirmed direct binding. In CRC and PC, high miR-181c/d-5p expression correlates with low RBM47 expression (14, 15). In addition, RBM47 binds and stabilizes the long non-coding RNA (lncRNA) SNHG5 (small nucleolar RNA host gene 5), which recruits the deubiquitinase ubiquitin-specific peptidase 21 (USP21) to stabilize FOXO3 protein indirectly (10). In zebrafish cell lines, *rbm47* transcript levels begin to rise at 6 hours after polyinosinic:polycytidylic acid (poly I:C) transfection or spring viremia of carp virus (SVCV) infection and peak at 24 hours (6).

### 1.3 Physiological functions

#### 1.3.1 Embryonic development and stem cell differentiation

**Figure 2:**
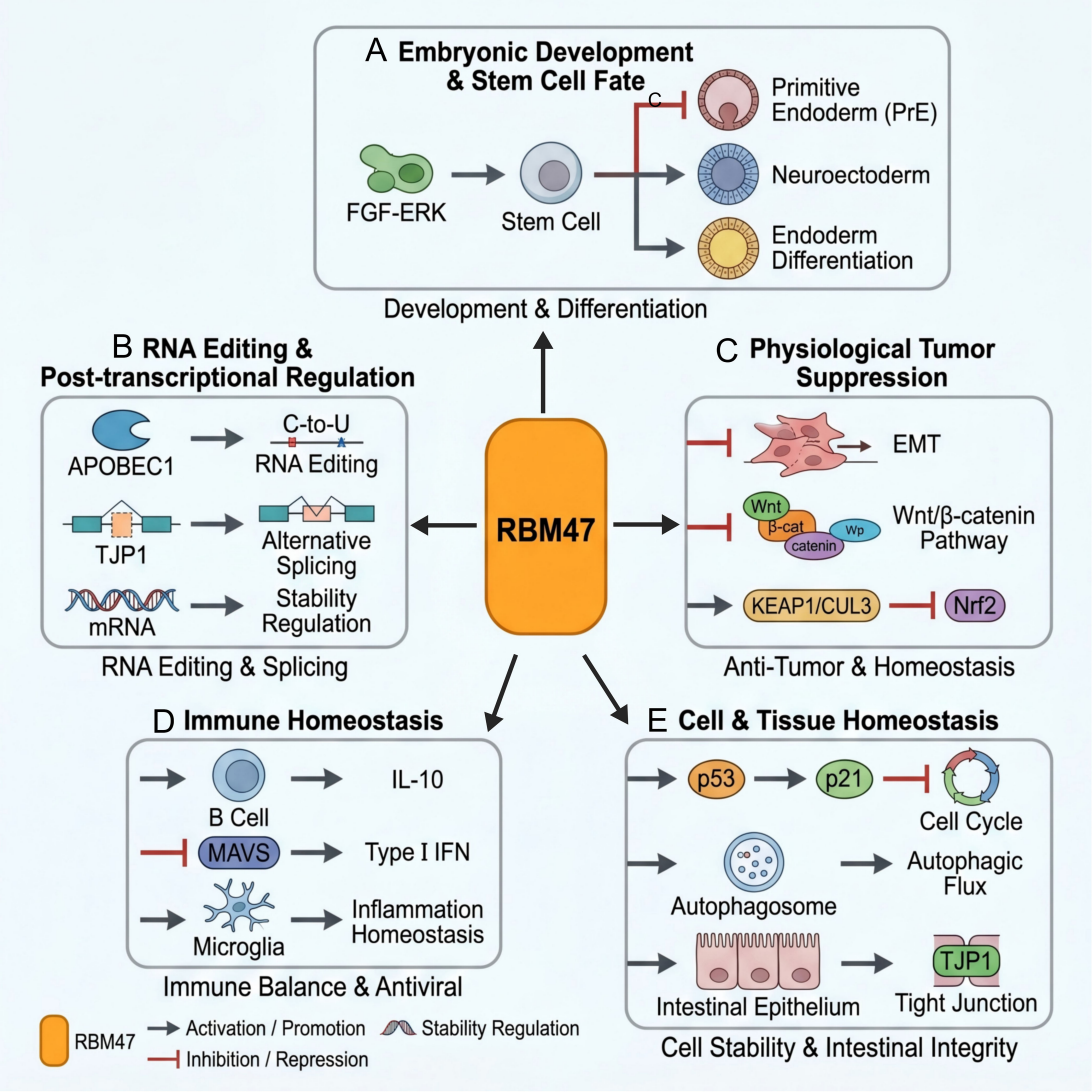
The core functional network of RBM47 in physiological homeostasis. RBM47 orchestrates a multifunctional regulatory network across embryonic development, post-transcriptional RNA metabolism, immune homeostasis, and tissue integrity. **(A)** In embryonic stem cells, RBM47 integrates with the FGF-ERK signaling axis to promote neuroectoderm and definitive endoderm differentiation while suppressing primitive endoderm (PrE) fate. **(B)** As a central post-transcriptional regulator, RBM47 assembles with APOBEC1 to drive C-to-U RNA editing, modulates alternative splicing (e.g., *TJP1* exon 20 inclusion), and predominantly enhances target mRNA stability. **(C)** In tumor suppression, RBM47 restrains malignant progression by inhibiting EMT, the Wnt/β-catenin pathway, and the KEAP1-CUL3-Nrf2 antioxidant axis. **(D)** In immune homeostasis, RBM47 promotes IL-10 production in regulatory B cells and negatively regulates MAVS-mediated type I interferon signaling in zebrafish; its expression in microglia participates in inflammatory modulation. **(E)** RBM47 maintains cell and tissue homeostasis by activating the p53-p21 cell cycle checkpoint, enhancing autophagic flux, and preserving intestinal epithelial tight junction integrity via *TJP1* splicing regulation.

RBM47 is a key regulatory factor for early vertebrate development (1). In zebrafish, *rbm47* deficiency causes *wnt8a* upregulation and impairs embryonic head development, producing headless or small-head phenotypes (16, 17). In mice, homozygous inactivation of *Rbm47* causes perinatal lethality and fetal resorption after embryonic day 10; retention of one functional allele is required for embryonic survival and postnatal growth (4, 18, 19). Mouse embryonic stem cell (ESC) studies show that RBM47 loss does not affect pluripotency maintenance (Oct4, Nanog, and Sox2 expression remain unchanged) but impairs multi-lineage differentiation potential; knockdown cells form teratomas of markedly reduced volume (5, 20). RBM47 is required for normal differentiation of the neuroectoderm and definitive endoderm; its loss downregulates neural precursor markers (Pax6, Nestin) and causes biased upregulation of extraembryonic endoderm markers (Gata4, Gata6, Sox17) (21, 22). RBM47 suppresses primitive endoderm (PrE) fate through regulation of the fibroblast growth factor-extracellular signal-regulated kinase (FGF-ERK) signaling pathway; knockdown increases the proportion of GATA4-positive PrE-like cells from approximately 2.2% to approximately 6% (23, 24) (**Figure 2**).

#### 1.3.2 RNA editing and post-transcriptional regulation

RBM47 is a critical cofactor for mammalian APOBEC1 (apolipoprotein B mRNA editing enzyme catalytic subunit 1)-mediated C-to-U RNA editing. It can replace A1CF (APOBEC1 complementation factor) to form a functional editosome with APOBEC1 (1, 5). This editosome is responsible for cytidine-to-uridine editing of multiple target RNAs, including apolipoprotein B (*Apob*) mRNA; editing events occur mainly in the 3′-UTR and affect mRNA splicing patterns, translation efficiency, and stability (5, 25). Conditional knockout studies show that intestine-specific deletion of *Rbm47* abolishes editing activity for most targets, highlighting the dominant role of RBM47 in intestinal RNA editing (26).

In alternative splicing, RBM47 recognizes specific sequence motifs in pre-mRNA to modulate splicing patterns. In *TJP1* (tight junction protein 1, also known as ZO-1) pre-mRNA, RBM47 recognizes the “UGCAUG” motif in the intron downstream of exon 20 and promotes exon 20 inclusion, generating the TJP1-α+ isoform (27, 28).

In mRNA stability control, RBM47 enhances stability by binding to the 3′-UTR of target mRNAs. Identified physiological targets include interleukin-10 (*Il10*) in B cells and interferon alpha and beta receptor subunit 1 (IFNAR1) (29, 30). In pathological states, RBM47 also regulates the stability of *AXIN1* (axis inhibition protein 1), *PTEN* (phosphatase and tensin homolog), *PDIA6* (protein disulfide isomerase family A member 6), *cGAS* (cyclic GMP-AMP synthase), *KEAP1/CUL3* (kelch-like ECH-associated protein 1/Cullin 3), and *TRAF6* (TNF receptor associated factor 6) mRNAs (9, 12, 15, 31, 32).

#### 1.3.3 Immune homeostasis maintenance

In B cells, RBM47 participates in immune regulation. *Rbm47* expression is markedly higher in regulatory B lymphocytes than in interleukin-10 (IL-10)-negative B cells. RBM47 binds the AU-rich element in the 3′-UTR of *Il10* mRNA and positively regulates its stability, thereby promoting IL-10 production and immunosuppressive function (29).

In antiviral immunity, the role of RBM47 is species-specific. In zebrafish, RBM47 interacts with MAVS and promotes lysosome-dependent degradation of MAVS (sensitive to blockade by the lysosome inhibitors ammonium chloride or chloroquine), thereby inhibiting type I interferon (IFN) expression and preventing excessive inflammatory damage (6). In mammalian cells, RBM47 stabilizes *IFNAR1* mRNA and enhances the host response to type I interferons (30) (**Table 1**).

**Table 1:** Core molecular functions and downstream target spectrum of RBM47.

Functional category	Molecular mechanism	Direct target genes / interacting partners	Cellular / tissue context	Refs
C-to-U RNA editing	Cofactor for APOBEC1-mediated editosome assembly	APOBEC1, Apob mRNA, multiple 3′-UTR substrates	Intestine, liver	(1, 5, 25, 26)
Alternative splicing	Recognition of intronic “UGCAUG” motif	TJP1 (ZO-1 pre-mRNA), EMT-associated transcripts	Colorectal epithelium, EMT models	(27, 28)
mRNA stabilization (physiological)	3′-UTR binding (AU-rich elements)	Il10, IFNAR1	Regulatory B cells, mammalian immune cells	(29, 30)
mRNA stabilization (pathological)	3′-UTR binding and half-life extension	AXIN1, PTEN, PDIA6, cGAS, TRAF6, KEAP1/CUL3	NSCLC, CRC, pancreatic cancer, glioma, POCD models	(9, 12, 15, 31, 32, 62)
lncRNA scaffolding	Direct binding and stabilization	SNHG5, HOXB-AS1	PTC, RCC	(2, 10)
Innate immune regulation	MAVS interaction and lysosomal targeting	MAVS, MITA (STING)	Zebrafish (viral infection models)	(6)
Developmental regulation	FGF-ERK pathway modulation	wnt8a	Zebrafish embryogenesis	(16, 17)

FOXO3, ATG3, and ATG5 are not listed as direct targets because RBM47 regulates them indirectly via the SNHG5–USP21–FOXO3 axis. Only direct interactors are placed in the Direct Target Genes column to maintain scientific accuracy.

### 1.4 Structural features of RBM47 and its position within the RBM family

#### 1.4.1 Sequence composition and structural prediction of RBM47

Based on amino acid sequence analysis, RBM47 is classified as an RNA-binding protein containing three tandem RRM domains (1). To date, no high-resolution experimental structure of full-length RBM47 or any individual RRM domain has been reported by X-ray crystallography, nuclear magnetic resonance (NMR), or cryo-electron microscopy (Cryo-EM). Nevertheless, existing functional studies have indirectly reflected the binding activity of its domains: for example, RBM47 binds AU-rich elements in the 3′-UTR of target mRNAs (e.g., *IFNAR1*) to regulate mRNA stability (30), and in glioma RBM47 participates in a feedback loop with NF-κB through its interaction network (12). However, the key residues at the RBM47-RNA binding interface, conformational dynamics, and the spatial arrangement of the three RRM domains in three dimensions remain uncharacterized by direct structural biology. In recent years, bioinformatic models such as AlphaFold2 have advanced protein folding prediction, but the predicted fine three-dimensional structure and potential intrinsically disordered regions (IDRs) of RBM47 await experimental validation.

#### 1.4.2 Structural insights from other RBM family members

Compared with the limited structural data for RBM47, high-resolution structures of several other RBM family members have been resolved, providing a reference for understanding RRM diversity and inferring the potential spatial features of RBM47.

Isolated RRM domains commonly adopt the canonical βαββαβ fold. For example, the N-terminal RRM of RBM3 was resolved by solution NMR in this fold, and its C-terminus was found to be rich in RGG/YGG motifs and to exhibit an IDR (33). The RRM crystal structure of Fox-1 (1.8 Å) illustrated the role of hydration dynamics in RNA recognition (39). The RRM domain of RBM7 can adopt a cyclic pentameric conformation under specific conditions (38).

Among multi-RRM proteins, RBM45, which shares the “three-RRM” architecture with RBM47, has had its N-terminal tandem RRM1–2 crystal structure resolved in complex with GAC-containing nucleic acids. The structure shows that the two RRMs form an antiparallel arrangement and recognize RNA through aromatic residues and arginine (36). The C-terminal RRM3 of RBM45 has also been shown to bind the GACG sequence specifically (37, 40). In addition, the tandem RRM1–2 of RBM39 matches experimental secondary-structure predictions (34), and its RRM2 domain was resolved at 2.3 Å in a complex with the DCAF15 E3 ubiquitin ligase and the small-molecule drug Indisulam, revealing how Indisulam embeds into the protein interface to mediate RBM39 degradation (35).

#### 1.4.3 Structural commonalities, differences, and theoretical basis for targeted drug development

RBM47 shows both commonalities and differences with the above family members in structural composition and functional association. These comparisons provide a theoretical reference for subsequent drug development.

Structural and functional commonalities: Family members use evolutionarily conserved RRMs as basic units to participate in RNA metabolism. Just as RBM39 participates in tumor progression through the Wnt/β-catenin pathway (41) and RBM45 abnormal aggregation is associated with neurodegenerative diseases (36, 37), RBM47 participates in the regulation of NSCLC cell properties and metastasis through AXIN1 and the Wnt pathway (31, 42).

Structural and mechanistic differences: Although RBM47 and RBM45 both contain three RRMs, the spatial synergy and substrate specificity of each RRM in RBM45 are supported by structural data (36, 37, 40), whereas whether allostery exists among the three RRMs of RBM47 remains unclear. In addition, whether RBM47 possesses a C-terminal disordered regulatory element similar to that of RBM3 (33) or a degron similar to that of RBM39 (35) has not been determined experimentally. Current functional studies of RBM47 focus mainly on cellular phenotypes and molecular-level mechanisms; atomic-level mechanisms require further investigation.

Theoretical basis for drug development: Progress in structural characterization of family members provides exploratory directions for RBM47-targeted intervention. First, referring to the well-defined RNA recognition pockets in the RBM45/Fox-1 structures, future efforts could attempt high-throughput virtual screening to develop small-molecule inhibitors that block the RBM47-specific mRNA binding interface. Second, drawing on the mechanism by which Indisulam acts as a “molecular glue” to target RBM39 and recruit an E3 ubiquitin ligase for degradation (35), the design of PROTACs (proteolysis targeting chimeras) or molecular glue drugs against specific RBM47 domains may represent a potential strategy for diseases associated with abnormal RBM47 expression. The structural and functional differences between RBM47 and representative RBM family members are summarized in **Table 2**.

**Table 2:** Structural and functional comparison of RBM47 with representative RBM family members.

Protein	RRM number and structural features	Functional associations (strictly evidence-based)	Key evidence types
RBM47	Three tandem RRMs (sequence-predicted); no experimentally resolved structure currently available	(1) RNA metabolism: Cofactor for APOBEC1-mediated C-to-U RNA editing; regulates alternative splicing (e.g., *TJP1* exon 20 inclusion); stabilizes target mRNAs (e.g., *AXIN1*, *PTEN*, *Il10*, *IFNAR1*). (2) Tumor suppression: Downregulated in colorectal cancer, non-small cell lung cancer, renal cell carcinoma, papillary thyroid carcinoma, and breast cancer; suppresses proliferation, EMT, and stemness via Wnt/β-catenin, PI3K-AKT, Nrf2, and p53 pathways. (3) Oncogenic roles: Upregulated in glioma and pancreatic cancer; promotes M2 macrophage polarization (glioma) and NK cell dysfunction/PD-L1–mediated immune evasion (pancreatic cancer). (4) Immune modulation: Enhances IL-10 production in regulatory B cells; negatively regulates MAVS-mediated antiviral signaling in zebrafish. (5) Autophagy: Indirectly activates *ATG3/ATG5* transcription via the SNHG5–USP21–FOXO3 axis.	Cell-based assays Animal models Clinical cohorts
RBM39	Tandem RRM1–2 (structurally validated); RRM2 participates in the Indisulam–DCAF15 molecular glue degradation complex	Splicing regulation; promotes cholangiocarcinoma progression via Wnt/β-catenin; susceptible to targeted proteolysis by Indisulam.	Structural biology Cell experiments
RBM45	Three RRMs: N-terminal RRM1–2 in antiparallel arrangement recognizing GAC; RRM3 specifically recognizes GACG	Abnormal aggregation associated with ALS/FTLD; computational prediction suggests m^6^A-modified RNA recognition (Ref. 40).	Structural biology Cell experiments
RBM3	Single RRM (NMR-resolved, βαββαβ topology); C-terminal RGG/YGG intrinsically disordered region	Functional roles not expanded in the present text; structural features provide a folding paradigm reference for the RRM family.	Structural biology NMR spectroscopy

### 1.5 Literature search and selection criteria

This narrative review was conducted through systematic searches of PubMed, Web of Science, and Google Scholar (last search: April 2026). The primary search terms were combinations of the following: “RBM47”, “RNA-binding motif protein 47”, “cancer”, “tumor”, “glioma”, “pancreatic cancer”, “colorectal cancer”, “non-small cell lung cancer”, “NSCLC”, “papillary thyroid carcinoma”, “renal cell carcinoma”, “postoperative cognitive dysfunction”, “inflammatory bowel disease”, “antiviral immunity”, “MAVS”, “cGAS-STING”, “alternative splicing”, “mRNA stability”, and “epithelial-mesenchymal transition”. Inclusion criteria: peer-reviewed original research articles, clinical studies with human tissue data, and authoritative reviews published in English. Exclusion criteria: non-peer-reviewed preprints without full experimental data, conference abstracts lacking methodological detail, and studies without accessible full text. Reference lists of retrieved articles were manually screened for additional relevant studies. No prospective clinical trials or interventional studies targeting RBM47 were identified.

## 2 Roles and molecular mechanisms of RBM47 in disease

The mechanistic and clinical evidence summarized in this review differs substantially in experimental rigor, sample size, model relevance, and proximity to clinical application. To provide a transparent interpretive framework, we classified the available evidence into four tiers:Level I – Evidence from prospective clinical trials, multi-center prospective cohorts, or biomarkers validated in independent prospective studies. No Level I evidence for RBM47 currently exists; Level II – Evidence from: (a) single-center retrospective clinical cohorts with human tissue data; (b) public database mining (TCGA, CGGA, GEO, REMBRANDT) with correlative findings; (c) conditional knockout or transgenic animal models with phenotype rescue; or (d) mechanistic rescue experiments demonstrating causality *in vivo*; Level III – Evidence from: (a) *in vitro* cell-line studies with mechanistic insight; (b) xenograft models without immune-competent validation; (c) bioinformatic analyses interpreted as therapeutic predictiveness without pharmacodynamic validation; or (d) cross-species models (e.g., zebrafish) without independent mammalian validation; Level IV – Evidence based on: (a) in silico predictions without functional testing; (b) indirect pathway inferences; (c) correlation interpreted as therapeutic predictiveness; or (d) speculative combination therapy hypotheses without combination experimental data. This grading scheme is applied throughout the disease-specific sections and summarized in **Table 3**.

**Table 3:** Expression patterns, functional polarization, and clinicopathological correlations of RBM47 in human diseases.

Disease category	Disease	Expression pattern	Functional polarization	Core signaling axis / mechanism	Key clinicopathological correlation	Evidence type	Evidence level and translational readiness	Refs
Neoplastic diseases	Glioma	Significantly upregulated	Oncogenic	RBM47–NF-κB positive feedback; TRAF6/IL-1A/IL-1B stabilization; EMT activation; M2 macrophage polarization	Mesenchymal subtype-specific; correlates with WHO grade, IDH-wildtype, and poor OS (CGGA: HR = 1.047; TCGA: HR = 1.334)	Cell / Animal / Clinical cohort	Level II (retrospective bioinformatics: CGGA/TCGA/REMBRANDT; single-center IHC). Level III (cell-line mechanistic studies: U87, U251). Critical gap: No specific RBM47 inhibitor; M2 polarization shown only in mouse models; no prospective biomarker validation.	(12, 72, 73)
	Papillary thyroid carcinoma	Significantly downregulated	Tumor-suppressive	RBM47–SNHG5–USP21–FOXO3 autophagy axis; proliferation inhibition	Low expression correlates with larger tumor volume (P = 0.021) and advanced TNM stage (P = 0.006); overexpression reduces xenograft volume by ~60%	Cell / Animal / Clinical cohort	Level II (single-center retrospective cohort, n=100). Level III (xenograft model, n=5/group). Critical gap: No multi-center independent validation; no prospective cohort.	(10)
	Non-small cell lung cancer	Downregulated (heterogeneous; protein-level) / upregulated in LUAD vs LUSC (mRNA-level)	Tumor-suppressive	AXIN1/Wnt/β-catenin inhibition; KEAP1-CUL3-Nrf2 axis; PD-L1 mRNA destabilization	Low expression is an independent poor prognostic factor for OS (HR = 2.716) and RFS; high expression reduces PD-L1 mRNA in cell lines	Cell / Animal / Clinical cohort	Level II (single-center retrospective cohorts, n=136/64 (31); GEO database mining (90)). Level III (cell-line mechanistic studies (32,42)). Critical gap: Expression trends contradictory across IHC (31) vs. GEO (90); no multi-center validation; PD-L1 regulation demonstrated at mRNA level only.	(31, 32, 42, 54)
	Breast cancer	Downregulated (promoter methylation)	Tumor-suppressive	EMT and cancer stem cell inhibition; metastasis suppression	Low expression significantly correlates with lymph node/distant metastasis and poor survival	Clinical samples / Cell experiments	Level II (single-center retrospective analysis). Level III (doxycycline-inducible rescue in cell lines). Critical gap: No prospective validation; no clinical trial data.	(3)
	Pancreatic cancer	Significantly upregulated	Oncogenic	PDIA6 stabilization; CTTN/F-actin-mediated NK cell dysfunction; PD-L1 upregulation; metabolic reprogramming	High expression correlates with advanced T stage and a "cold" tumor microenvironment (low NK infiltration)	Cell / Animal / Clinical samples	Level II (single-center retrospective cohort). Level III (subcutaneous xenograft, n≈5–6). Critical gap: No PDX/organoid validation; NK dysfunction shown only *in vitro*; no immune-competent model.	(15)
	Colorectal cancer	Significantly downregulated (promoter hypermethylation)	Tumor-suppressive	EMT-associated alternative splicing network; PI3K-AKT pathway inhibition and apoptosis regulation; intestinal stemness modulation	CMS4 mesenchymal subtype marker; promoter methylation in liver metastases; low expression predicts shortened OS (P = 0.0071) and RFS (P = 0.0088)	Cell / Animal / Clinical cohort	Level II (TCGA & multi-cohort retrospective mining). Level III (enteroid validation for intestinal phenotypes (63); 5-Aza/TSA *in vitro* only). Critical gap: No prospective cohort; promoter methylation threshold not validated in liquid biopsy; 5-Aza/TSA combination only in cell lines.	(13, 62, 63)
	Renal cell carcinoma	Significantly downregulated	Tumor-suppressive	RBM47–HOXB-AS1–p53 axis; p53 nuclear translocation; enhanced sunitinib sensitivity	High expression is an independent favorable prognostic factor for OS (HR = 0.41, P = 4.4 × 10^-8^); low expression linked to chemoresistance	Cell / Animal / Clinical cohort	Level II (single-center retrospective cohort, n=40). Level III (subcutaneous xenograft, n=5). Critical gap: No multi-center validation; no prospective biomarker trial.	(2)
Non-neoplastic disorders	Postoperative cognitive dysfunction	Significantly upregulated	Pathogenic driver	RUNX1–RBM47–cGAS–STING–MEF2C–GPX4 axis; cGAS stabilization; neuronal ferroptosis	Hippocampal microglial expression; correlates with cognitive impairment severity; RUNX1 co-expression (r = 0.88)	Animal model	Level III (C57BL/6J mouse model only). Critical gap: No human biomarker data; no pharmacodynamic validation in surgical patients; preclinical intervention stage only.	(9)
	Inflammatory bowel disease / CAC	Conditionally lost	Dual regulator	Acute phase: FNDC5/NRF2 and IL-33/AREG activation (protective); Chronic phase: long-term loss promotes spontaneous tumorigenesis	Short-term deletion: colitis resistance; long-term deletion: spontaneous intestinal polyps (11/12 vs. control 1/13)	Animal model	Level II (conditional knockout mouse model with phenotype rescue). Level III (enteroid/colonoid validation). Critical gap: No human IBD cohort data; acute protection vs. chronic carcinogenesis balance unknown in clinical settings.	(63)
	Viral infection & innate immunity	Upregulated upon infection	Negative immune regulator	MAVS lysosomal degradation; MAVS–MITA interaction disruption; IRF3/IRF7 dephosphorylation	Enhanced viral replication; dampened type I interferon response	Cell / Animal	Level III (zebrafish and cell-line models). Critical gap: No human clinical infectious disease cohort; functional inversion between zebrafish and mammals complicates extrapolation.	(6)

### 2.1 Neoplastic diseases

#### 2.1.1 Solid tumors in which RBM47 exerts tumor-suppressive function

RBM47 exerts tumor-suppressive effects in multiple solid tumors. Its downregulation or loss correlates with tumor progression and poor prognosis. Common mechanisms include: suppression of Wnt/β-catenin and PI3K-AKT pathways through stabilization of negative regulator mRNAs; inhibition of epithelial-mesenchymal transition (EMT) through alternative splicing regulation; and promotion of cell death through activation of p53 or autophagy pathways.

##### 2.1.1.1 Papillary thyroid carcinoma

PTC is the most common endocrine malignancy, accounting for approximately 60-70% of all thyroid cancers. Most patients have a favorable prognosis and are cured by surgery and radioactive iodine therapy, but approximately 10-20% develop an aggressive phenotype characterized by neurovascular invasion, lymph node metastasis, recurrence, and poor response to conventional therapy (43–49). Preclinical studies indicate that RBM47 exerts a tumor-suppressive effect in PTC, and its loss is an important driver of malignant progression. Functional experiments show that RBM47 overexpression suppresses PTC cell proliferation, whereas knockdown promotes proliferation (10).

At the molecular level, RBM47 constructs the SNHG5/USP21/FOXO3 positive feedback axis to suppress proliferation and activate autophagy: RBM47 binds and stabilizes the lncRNA SNHG5; SNHG5 recruits the deubiquitinase USP21; USP21 inhibits proteasomal degradation of FOXO3 through deubiquitination and promotes FOXO3 nuclear translocation. Nuclear FOXO3 binds the *ATG3* and *ATG5* promoters directly to activate autophagy gene transcription and promote autophagosome formation, and also binds the RBM47 promoter to activate its transcription, forming a positive feedback loop (10). In PTC, this loop is disrupted because of low RBM47 and SNHG5 expression, resulting in insufficient autophagy and excessive cell proliferation. *In vivo* experiments show that RBM47 overexpression suppresses growth of subcutaneous xenografts in nude mice, and co-knockdown of SNHG5 partially reverses this tumor-suppressive effect (10). These findings provide Level II/III preclinical evidence for RBM47 as a tumor suppressor in PTC.

##### 2.1.1.2 Non-small cell lung cancer

NSCLC is the malignancy with the highest incidence and mortality worldwide, accounting for approximately 85% of all lung cancer cases. It consists mainly of lung adenocarcinoma and lung squamous cell carcinoma. The 5-year survival rate for advanced patients remains below 20%; tumor metastasis, recurrence, and persistence of cancer stem cells are the main causes of treatment failure (50–53).

The expression trend of RBM47 in NSCLC remains controversial across studies. In a retrospective cohort of 136 NSCLC patients using immunohistochemistry, RBM47 protein was significantly downregulated in tumor tissues compared with adjacent normal tissues, and low expression correlated with advanced TNM stage and poor prognosis (31). However, divergent mRNA expression patterns have been reported by independent GEO dataset analyses, including higher RBM47 mRNA in NSCLC tissues than in paired normal tissues and significant elevation in lung adenocarcinoma compared with lung squamous cell carcinoma (54). These inconsistencies suggest that detection platform (IHC versus microarray), antibody specificity, and histological subtype contribute to observed heterogeneity. The functional and protein-level evidence generally supports a tumor-suppressive role for RBM47 in NSCLC, but its mRNA expression patterns across subtypes and cohorts are not uniform.

The established RBM47 regulatory mechanisms in NSCLC include: (1) AXIN1/Wnt/β-catenin pathway inhibition: RBM47 binds directly to the 3′-UTR of *Axin1* mRNA to enhance its stability, maintain β-catenin destruction complex function, promote β-catenin proteasomal degradation, inhibit Wnt pathway activation and EMT, and reduce metastasis. (2) Cancer stem cell property inhibition: RBM47 expression correlates negatively with stemness markers Prominin-1 (CD133) and Thy-1 cell surface antigen (CD90); RBM47 loss enhances sphere-forming capacity and tumorigenicity of cancer stem cells through Wnt pathway activation, and this effect is reversed by Wnt inhibitors. (3) Immune microenvironment regulation: RBM47 binds the 3′-UTR of programmed death-ligand 1 (*PD-L1*) mRNA to promote its degradation, downregulates PD-L1 protein in tumor cells, and enhances T cell-mediated tumor killing *in vitro*. (4) Nrf2 antioxidant pathway regulation (the main mechanism in lung adenocarcinoma): in this subtype, RBM47 suppresses tumor growth through the KEAP1-Cullin 3 (CUL3)-Nrf2 axis. RBM47 upregulates KEAP1 and CUL3 expression through post-transcriptional regulation. KEAP1 is the substrate adaptor of the CUL3 ubiquitin ligase complex; it recognizes and binds Nrf2, promoting its K48-linked polyubiquitination and proteasomal degradation (31, 32, 42).

High RBM47 expression accelerates Nrf2 degradation, inhibits Nrf2-mediated antioxidant stress responses and cell proliferation signals, blocks expression of downstream antioxidant genes (e.g., heme oxygenase-1 [*HO-1*] and NAD(P)H quinone oxidoreductase 1 [*NQO1*]), and increases tumor cell sensitivity to oxidative stress. Cell and animal experiments confirm that RBM47 overexpression suppresses proliferation and colony formation of lung adenocarcinoma cells, and re-expression of RBM47 inhibits xenograft growth. RBM47 loss or low expression causes decreased KEAP1 and CUL3 levels, Nrf2 accumulation and persistent activation, and tumor progression (32). These mechanisms are supported by Level II/III evidence.

##### 2.1.1.3 Breast cancer

BC is the most common malignancy in women. According to molecular characteristics, it is classified into luminal, HER2-positive, and basal-like (triple-negative) subtypes. Tumor metastasis is the main cause of patient death and involves a complex process including detachment from the primary lesion, vascular invasion, and distant colonization (55–58). Preclinical evidence indicates that RBM47 is a key suppressor of BC metastasis; its loss provides selectable metastatic traits. Through its RNA-binding activity, RBM47 regulates the stability or translation efficiency of multiple target mRNAs involved in cell adhesion, migration, and invasion, thereby inhibiting EMT and acquisition of stem cell-like properties (3). When RBM47 expression is lost or downregulated, pro-metastatic mRNAs gain increased stability or translation efficiency, producing a more invasive phenotype. Clinicopathological analysis from a single retrospective cohort shows that low RBM47 expression correlates with lymph node metastasis, distant metastasis, and poor prognosis in BC. Its expression is often downregulated by promoter methylation or transcriptional suppression, and this epigenetic silencing is an important mechanism promoting tumor progression (3). These findings constitute Level II/III evidence.

##### 2.1.1.4 Colorectal cancer

CRC is the third most common malignancy worldwide. Its development follows the “adenoma-carcinoma” sequence and involves mutations in *APC*, *KRAS*, *TP53*, and epigenetic alterations. Tumor metastasis is the main cause of patient death, and understanding its molecular mechanisms is critical for developing new therapeutic strategies (59–61). Functional validation shows that RBM47 knockdown markedly enhances migration and invasion of CRC cells and promotes lung metastasis in a tail vein injection model (62); in *ApcMin/+* mice, *Rbm47* loss cooperates with *Apc* mutation to promote small intestinal polyp growth and accelerate adenoma progression (63).

RBM47 exerts tumor-suppressive effects in CRC through multiple mechanisms: (1) EMT regulation: RBM47 is a core regulator of the EMT-associated alternative splicing network. Its downregulation during CRC progression causes accumulation of pro-metastatic splice variants and enhances tumor cell migratory capacity. This regulatory network cross-talks with EMT key transcription factors (SNAIL, zinc finger E-box binding homeobox [ZEB] family) to determine tumor cell phenotypic plasticity (62). (2) TJP1 splicing and proliferation signal regulation: RBM47 regulates *TJP1* exon 20 splicing. Loss of RBM47 reduces the TJP1+E20 splice variant, which cannot bind and inhibit ZONAB (Y-box binding protein 3, YBX3), leading to increased ZONAB nuclear translocation and activation of pro-proliferative target gene transcription. Concurrently, RBM47 loss causes overactivation of Wnt/β-catenin and mitogen-activated protein kinase (MAPK) signaling, driving cell cycle progression and abnormal proliferation (27, 62). (3) Stem cell property regulation: Low RBM47 expression upregulates intestinal stem cell markers (Lgr5, Lrig1, Smoc2), enhances organoid formation efficiency and proliferation capacity of intestinal stem cells, and promotes cancer stem cell properties (63). (4) PI3K/AKT pathway inhibition: RBM47 binds the 3′-UTR of *PTEN* directly to enhance its mRNA stability, thereby inhibiting PI3K/AKT pathway activation. RBM47 downregulation causes increased p-PI3K and p-AKT levels, promoting tumor proliferation and survival (62). (5) Apoptosis regulation: RBM47 upregulates the pro-apoptotic gene caspase-3 (*CASP3*) and downregulates anti-apoptotic genes cellular communication network factor 1 (*CCN1*) and activating transcription factor 5 (*ATF5*), promoting apoptosis and inhibiting proliferation in CRC cells (62).

The evidence for RBM47 in CRC includes Level II findings (TCGA database mining and retrospective cohort analyses) and Level III findings (cell-line mechanistic studies, xenograft models, and enteroid validation). The promoter methylation association with liver metastasis was derived from a retrospective analysis of 43 patients and awaits prospective validation (13).

##### 2.1.1.5 Renal cell carcinoma

RCC is one of the most lethal malignancies of the urinary system. More than 10% of patients have distant metastasis at diagnosis, and the 5-year survival rate for metastatic RCC is only 12%. Although targeted therapy has improved prognosis in some patients, most eventually develop drug resistance, and survival remains unsatisfactory (64–68). Preclinical studies indicate that RBM47 exerts a tumor-suppressive effect in RCC, and its loss is an important marker of tumor progression and poor prognosis. Clinical sample analysis from a single retrospective cohort (n = 40) shows that both RBM47 mRNA and protein levels are markedly lower in RCC tumor tissue than in adjacent normal tissue, and expression correlates negatively with tumor grade. High expression correlates with longer overall survival (OS) and disease-free survival and is an independent protective factor for RCC prognosis (2). Functional experiments confirm that RBM47 overexpression suppresses RCC cell proliferation, migration, and xenograft growth, whereas knockdown promotes these malignant phenotypes. At the molecular level, RBM47 activates the p53 signaling pathway through the “RBM47-HOXB-AS1-p53” axis: RBM47 binds the lncRNA HOXB cluster antisense RNA 1 (HOXB-AS1) directly, competitively disrupts the HOXB-AS1-p53 interaction, relieves the cytoplasmic retention of p53 by HOXB-AS1, promotes p53 nuclear translocation, and activates transcription of downstream tumor suppressor genes including cyclin-dependent kinase inhibitor 1A (*p21*), *GADD45A*, and *GADD45B*, thereby inducing cell cycle arrest and inhibiting proliferation (2). These findings constitute Level II/III evidence.

In summary, RBM47 exerts tumor-suppressive effects in the above solid tumors through convergent pathways. Its target mRNAs are mainly negative regulators (AXIN1, PTEN, KEAP1/CUL3) and pro-autophagy/pro-apoptotic molecules. Functional loss causes uncontrolled proliferation signaling, EMT activation, and enhanced metastatic capacity. The downregulation mechanisms differ among tumors and involve promoter methylation, miRNA suppression, or transcription factor regulation.

#### 2.1.2 Malignant tumors in which RBM47 exerts oncogenic function

In contrast to the above solid tumors, RBM47 is highly expressed in glioma and PC and exerts oncogenic effects through remodeling of the immune microenvironment and activation of intrinsic tumor malignant programs.

##### 2.1.2.1 Glioma

Glioma is the most common malignant tumor of the central nervous system. Glioblastoma accounts for approximately 80% of central nervous system malignancies and is the most aggressive subtype, with a median survival of less than 15 months (69, 70). Tumor-associated macrophage polarization toward the M2 phenotype in the tumor microenvironment is a key factor promoting glioma progression and immune suppression and is closely associated with poor patient prognosis (71). Preclinical evidence indicates that RBM47 is a key factor driving malignant progression of glioma and is markedly upregulated in glioma tissue. It exerts oncogenic effects through both tumor cell-intrinsic mechanisms and microenvironment-extrinsic regulation (72, 73): (1) Tumor cell-intrinsic regulation: RBM47 is specifically upregulated in the mesenchymal subtype of glioma and promotes tumor cell invasion through EMT program activation. Overexpression of RBM47 upregulates mesenchymal markers (Vimentin, CD44) and downregulates the epithelial marker E-cadherin, thereby enhancing tumor cell proliferation, migration, and invasion (74). (2) Immune microenvironment regulation: RBM47 is specifically enriched in M2 macrophages and promotes M2 polarization through the RBM47-NF-κB positive feedback loop: RBM47 binds the 3′-UTR of *TRAF6* mRNA directly to enhance its stability and activate NF-κB signaling; activated NF-κB in turn binds the RBM47 promoter to promote its transcription. Concurrently, RBM47 binds and stabilizes pro-inflammatory factor mRNAs (interleukin-1 alpha [*IL-1A*] and interleukin-1 beta [*IL-1B*]), promotes their secretion, and drives M2 macrophage polarization and recruitment in an autocrine/paracrine manner, constructing an immunosuppressive microenvironment (12). In addition, RBM47 expression correlates positively with inhibitory immune checkpoint molecules T-cell immunoglobulin and mucin-domain containing-3 (TIM-3) and programmed cell death protein 1 (PD-1), suggesting its participation in tumor immune evasion in preclinical models.

These findings are based on Level II evidence (retrospective bioinformatic cohorts: CGGA, TCGA, REMBRANDT) and Level III evidence (cell-line and xenograft mechanistic studies). No prospective clinical validation or immune-competent *in vivo* model data exist.

##### 2.1.2.2 Pancreatic cancer

PC is a highly lethal malignancy with an overall 5-year survival rate below 5%. It ranks seventh among cancer-related deaths worldwide. More than 80% of patients are diagnosed at an advanced stage and have lost the opportunity for radical surgery. PC is highly resistant to existing radiochemotherapy, and new therapeutic targets are urgently needed to improve patient prognosis (75, 76). Preclinical studies indicate that RBM47 exerts oncogenic functions in PC, and its high expression is a key factor driving malignant progression. Clinical sample analysis from a single retrospective cohort shows that RBM47 is markedly upregulated in PC tissue and correlates negatively with T stage and natural killer (NK) cell infiltration. Functional experiments confirm that RBM47 overexpression promotes PC cell proliferation, colony formation, and xenograft growth, whereas knockdown inhibits these malignant phenotypes (15).

At the molecular level, RBM47 promotes cell proliferation and immune evasion through the “RBM47-PDIA6-CTTN/F-actin/PD-L1” multi-tier signaling axis: (1) Maintenance of PDIA6 mRNA stability: RBM47 binds the 3′-UTR of *PDIA6* mRNA directly, prolongs its half-life, and upregulates PDIA6 protein. PDIA6 is the key downstream molecule mediating the oncogenic effect of RBM47. (2) Immune evasion regulation: On one hand, PDIA6 upregulation promotes cortactin (CTTN) expression; CTTN interacts with filamentous actin (F-actin) to promote its accumulation at the immunological synapse, obstructing NK cell cytotoxic granule release. On the other hand, the RBM47/PDIA6 axis upregulates PD-L1 protein, inhibits NK cell activation through the PD-1/PD-L1 pathway, and reduces NK cell killing of tumor cells. (3) Metabolic reprogramming: RBM47 knockdown markedly alters the metabolic profile of PC cells, affecting key metabolic pathways including glutathione metabolism, arginine biosynthesis, and the tricarboxylic acid (TCA) cycle, thereby supporting tumor progression and immune evasion through metabolic microenvironment regulation (15). These findings constitute Level II/III evidence. No patient-derived xenograft (PDX) or immune-competent model validation has been reported.

**Figure 3:**
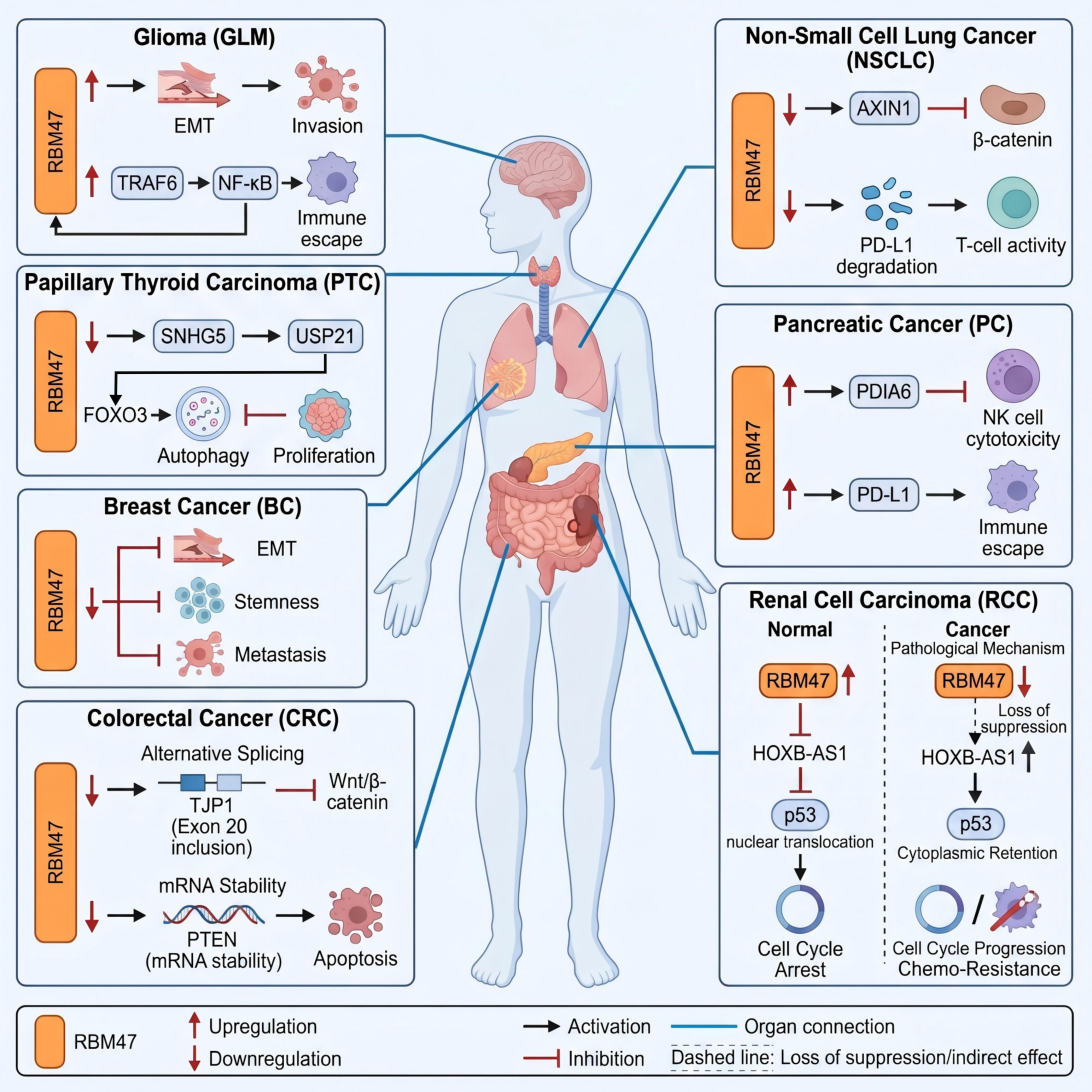
Bidirectional roles and context-dependent mechanisms of RBM47 in neoplastic diseases. RBM47 functions as a highly context-dependent "double-edged sword" across various malignancies. It acts as a potent tumor suppressor in non-small cell lung cancer (NSCLC), breast cancer (BC), and colorectal cancer (CRC) by inhibiting proliferation, metastasis, and oncogenic signaling (e.g., Wnt/β-catenin and EMT), and in papillary thyroid carcinoma (PTC) and renal cell carcinoma (RCC) by suppressing proliferation and malignant progression. Conversely, RBM47 serves as an oncogenic driver in glioma (GLM) and pancreatic cancer (PC): in GLM, it promotes invasion and M2 macrophage polarization to construct an immunosuppressive microenvironment; in PC, it drives NK cell dysfunction and PD-L1–mediated T-cell evasion.

In summary, RBM47 exerts oncogenic effects in glioma and PC through a dual mode of intrinsic malignant transformation of tumor cells and immune microenvironment suppression. The common features are the establishment of positive feedback loops to maintain high expression, and the construction of an immunosuppressive microenvironment through regulation of immune checkpoint molecules or macrophage polarization (**Figure 3**).

#### 2.1.3 Critical evaluation of preclinical evidence and unresolved controversies

##### 2.1.3.1 Expression discrepancies and methodological limitations

RBM47 expression trends in non-small cell lung cancer are inconsistent across studies. In a retrospective cohort of 136 NSCLC patients using immunohistochemistry, RBM47 protein was significantly downregulated in tumor tissues compared with adjacent normal tissues, and low expression correlated with advanced TNM stage and poor prognosis (31). However, an independent analysis of multiple GEO datasets (GSE27262, GSE7670, GSE19804) reported that RBM47 mRNA levels were higher in NSCLC tissues than in paired normal tissues (54). This discrepancy may reflect differences between protein-level (IHC) and mRNA-level (microarray) measurements, antibody specificity, or cohort heterogeneity. Notably, within the same GEO analysis, RBM47 expression was significantly higher in lung adenocarcinoma (LUAD) than in lung squamous cell carcinoma (LUSC) (GSE10245, GSE14814, GSE21933; P < 0.001) (54). Thus, while RBM47 is generally considered a tumor suppressor based on functional and protein-level evidence, its mRNA expression patterns across NSCLC subtypes and cohorts are not uniform, and any translational application must account for this heterogeneity.

##### 2.1.3.2 Functional duality and the missing molecular switch

RBM47 acts as a tumor suppressor in colorectal, renal, thyroid, and breast cancers, yet functions as an oncogene in glioma and pancreatic cancer. This dichotomy is plausibly explained by tissue-specific target mRNA repertoires—stabilizing negative regulators (AXIN1, PTEN, KEAP1) in suppressor contexts versus stabilizing pro-inflammatory or pro-metastatic mRNAs (TRAF6, PDIA6) in oncogenic contexts. However, what determines which target repertoire predominates in a given cell type—cofactor availability, subcellular localization, post-translational modifications, or microenvironmental signals—remains entirely unknown. This knowledge gap severely limits the rational design of RBM47-targeted interventions.

##### 2.1.3.3 Correlation versus therapeutic predictiveness

Multiple studies have reported positive correlations between RBM47 and PD-1, PD-L1, or TIM-3 expression, and these correlations have been interpreted as suggesting immunotherapy responsiveness. However, correlation in bulk tumor transcriptomes does not establish that RBM47 status predicts response to immune checkpoint inhibitors in treated patients. No pharmacodynamic or clinical outcome data from ICI-treated cohorts stratified by RBM47 expression have been published. Until such data exist, all immunotherapy-related claims remain speculative (Level IV evidence).

##### 2.1.3.4 Cross-species extrapolation risks

In zebrafish, RBM47 negatively regulates type I interferon production by promoting MAVS lysosomal degradation, whereas in mammalian cells, RBM47 stabilizes IFNAR1 mRNA and enhances antiviral responses. This functional inversion between lower vertebrates and mammals means that mechanistic insights derived from zebrafish models cannot be directly extrapolated to human diseases without independent mammalian validation. Similarly, the RUNX1-RBM47-cGAS-STING axis in postoperative cognitive dysfunction has been validated only in C57BL/6J mouse models; no human postoperative cerebrospinal fluid or brain tissue data exist to confirm this axis in clinical POCD (9).

### 2.2 Non-neoplastic disorders

#### 2.2.1 Postoperative cognitive dysfunction

POCD refers to cognitive impairment including memory decline, decreased attention, and impaired information processing that occurs after surgery. It is common in elderly surgical patients, reduces quality of life, increases medical costs, and markedly elevates the risk of Alzheimer’s disease and mortality. Although neuroinflammation is considered the core driver of POCD, its exact molecular mechanisms are not fully understood (77–79). Animal experiments suggest that RBM47 may participate in POCD pathogenesis: in a POCD mouse model, *Rbm47* mRNA and protein levels are markedly upregulated in the hippocampus, and RBM47 is expressed mainly in microglia, suggesting its participation in POCD pathogenesis through neuroinflammation regulation (9).

Existing studies suggest that the mechanism of RBM47 in POCD may involve the RUNX1-RBM47-cGAS-STING-MEF2C-GPX4 axis-mediated neuronal ferroptosis: (1) Transcriptional activation: anesthesia and surgical stress induce upregulation of RUNX1 in microglia; RUNX1 binds the *Rbm47* gene promoter directly and promotes RBM47 transcription and protein synthesis. (2) mRNA stabilization: RBM47 may participate in stability regulation of *cGAS* mRNA through its RRM domains and markedly enhances *cGAS* mRNA stability. (3) cGAS-STING pathway activation: upregulation of cGAS protein activates the STING (stimulator of interferon genes) signaling pathway and promotes release of inflammatory factors including tumor necrosis factor alpha (TNF-α), interleukin-6 (IL-6), and interferon-beta (IFN-β); this process is sensitive to blockade by the cGAS inhibitor RU.521. (4) Neuronal MEF2C-GPX4 axis inhibition: inflammatory factors released by microglia act on neurons and cause downregulation of MEF2C and GPX4; MEF2C transcriptionally activates GPX4 to inhibit ferroptosis. (5) Neuronal ferroptosis: GPX4 downregulation causes neuronal ferroptosis, characterized by lipid reactive oxygen species (ROS) accumulation and lipid peroxidation (9). These results suggest that RBM47 may activate microglial cGAS-STING inflammatory signaling through stabilization of *cGAS* mRNA, thereby inhibiting the neuronal MEF2C-GPX4 axis and participating in neuronal ferroptosis, ultimately promoting POCD in preclinical models. This mechanism is supported by Level III evidence (mouse models only); no human biomarker data exist.

#### 2.2.2 Inflammatory bowel disease and colitis-associated cancer

IBD (including Crohn’s disease and ulcerative colitis) is a chronic relapsing inflammatory disease of the intestine. Its global prevalence is rising continuously. Long-term chronic inflammation is the main risk factor for CAC, and the pathogenesis involves persistent oxidative stress, DNA damage, and abnormal immune response (80–84). RBM47 exhibits context-dependent bidirectional regulation in IBD: it exerts a protective effect in the acute inflammatory phase, but long-term loss induces tumorigenesis. In the acute inflammatory phase, intestine-specific knockout of RBM47 (*Rbm47*-IKO mice) shows markedly enhanced resistance to dextran sulfate sodium (DSS)-induced acute colitis, with improved survival, reduced weight loss, lower disease activity index, and improved histological damage (63). The protective mechanisms involve three pathways: first, activation of the FNDC5/Nrf2 antioxidant axis—RBM47 loss causes compensatory enhancement of *Fndc5* mRNA stability, elevated fibronectin type III domain containing 5 (FNDC5) activates the Nrf2 pathway, and upregulates antioxidant gene expression. Second, activation of the IL-33/AREG tissue protection pathway—*Rbm47*-IKO mice show upregulated interleukin-33 (IL-33) and its downstream targets amphiregulin (AREG) and interleukin-18 (IL-18), which promote tissue repair through activation of type 2 innate lymphoid cells (ILC2) and the AREG-EGFR (epidermal growth factor receptor) signal. Third, suppression of pro-inflammatory macrophage infiltration—RBM47 loss reduces infiltration of F4/80-positive macrophages in the colonic stroma and decreases pro-inflammatory factor expression, reducing the risk of crypt abscess formation (63).

In the azoxymethane (AOM)-DSS-induced CAC model, *Rbm47*-IKO mice show fewer colonic polyps and less dysplasia than controls. However, long-term RBM47 loss carries a pro-carcinogenic risk: 12-month-old *Rbm47*-IKO mice develop spontaneous polyps in the small intestine and colon under a regular diet (11/12 affected vs. 1/13 in controls), indicating that RBM47 is a necessary molecule for maintaining long-term genomic stability and suppressing spontaneous tumorigenesis (63). Overall, the role of RBM47 in IBD and CAC is context-dependent: acute loss of RBM47 exerts a protective effect through enhanced antioxidant and tissue repair responses, but under long-term chronic inflammation or aging, RBM47 loss causes uncontrolled proliferation and promotes spontaneous tumorigenesis. These findings are based on Level II evidence (conditional knockout mouse models with phenotype rescue) and Level III evidence (enteroid/colonoid validation). No human IBD cohort data exist.

#### 2.2.3 Viral infection and innate immune regulation

Viral infection is a core pathological stimulus triggering host innate immune response. After RNA virus invasion, RIG-I-like receptors (RLRs, including RIG-I and MDA5) recognize viral RNA and undergo conformational change. Through the caspase activation and recruitment domain (CARD), they bind the mitochondrial adaptor protein MAVS, initiating downstream signaling cascades that induce type I interferon production. However, excessive IFN response causes tissue damage and autoimmune pathology, so the organism requires precise negative feedback mechanisms to terminate immune responses in a timely manner (85–89). Functional validation shows that in zebrafish cells overexpressing RBM47, SVCV infection produces stronger cytopathic effects and higher viral titers; knockdown of RBM47 markedly upregulates IFN and the virus-induced gene 1 (VIG1), confirming that RBM47 weakens host antiviral defense through inhibition of MAVS function in zebrafish (6).

**Figure 4:**
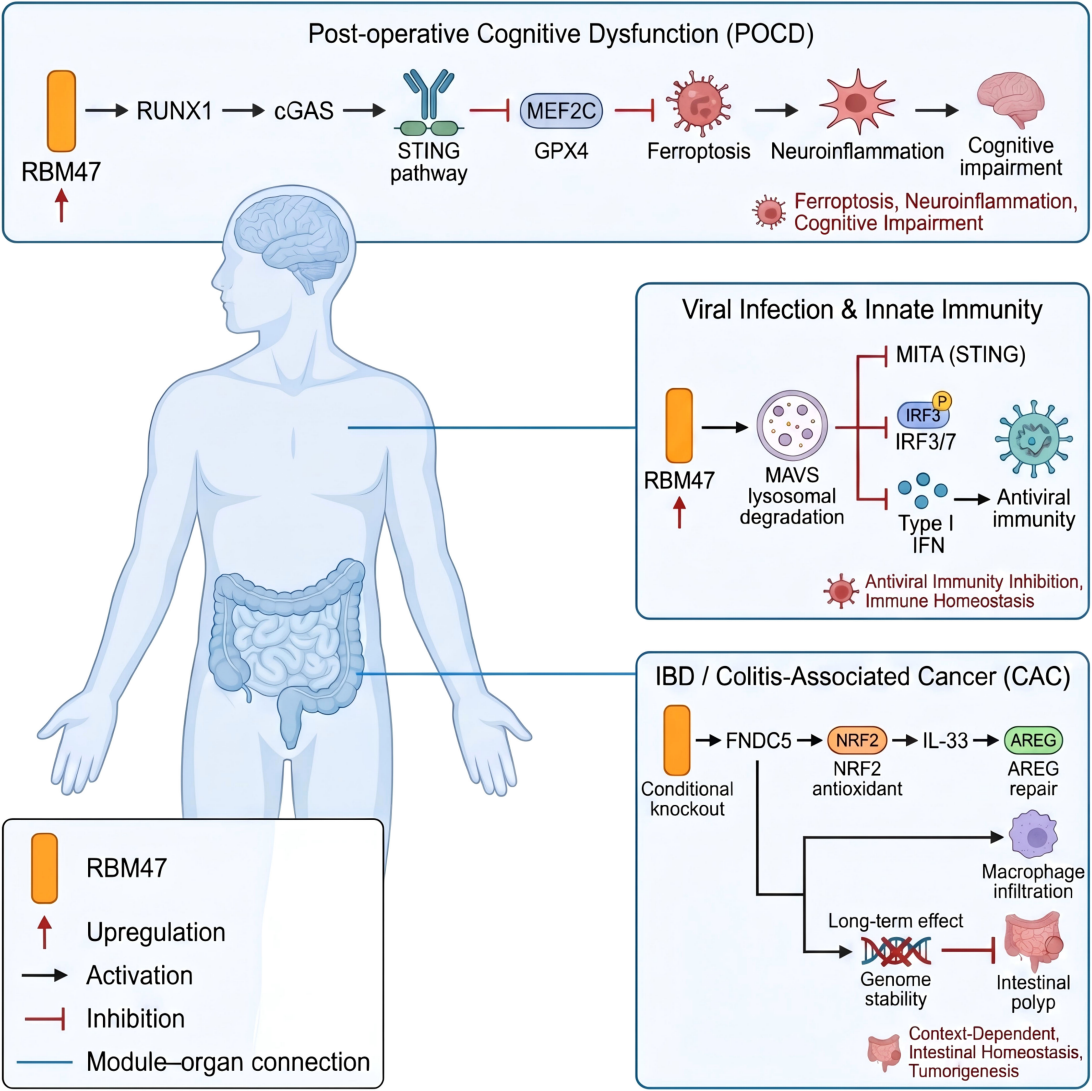
Pathophysiological regulatory mechanisms of RBM47 in non-neoplastic diseases. Aberrant RBM47 signaling drives the pathogenesis of various non-neoplastic and inflammatory disorders. In postoperative cognitive dysfunction (POCD), stress-induced RUNX1 upregulates RBM47, which stabilizes cGAS mRNA to trigger the STING pathway, ultimately leading to neuroinflammation and neuronal ferroptosis. In the context of viral infections, RBM47 functions as an immunological brake by promoting the lysosomal degradation of MAVS to maintain innate immune homeostasis. In inflammatory bowel disease (IBD) and colitis-associated cancer (CAC), RBM47 exhibits a stage-dependent dual role: its acute loss activates tissue repair and antioxidant responses, whereas its long-term deficiency compromises genomic stability and promotes spontaneous intestinal polyposis.

RBM47 is a key negative regulator of the RLR-MAVS signaling axis and inhibits antiviral signaling through three pathways: (1) Lysosome-dependent degradation of MAVS: RBM47 binds MAVS and alters its subcellular localization, transporting it from the mitochondrial outer membrane to the lysosomal region for degradation. This process is sensitive to blockade by lysosome inhibitors ammonium chloride and chloroquine but is unaffected by the proteasome inhibitor MG132 or the autophagy inhibitor 3-methyladenine (3-MA). (2) Disruption of the MAVS-MITA interaction: MAVS must recruit the endoplasmic reticulum adaptor protein MITA (mediator of IRF3 activation, also known as STING) to activate downstream TANK-binding kinase 1 (TBK1) and interferon regulatory factor 3/7 (IRF3/IRF7). RBM47 weakens this interaction in a dose-dependent manner through competitive binding or conformational change, blocking signal transmission downstream. (3) Inhibition of IRF3/IRF7 phosphorylation: MAVS degradation and ineffective MITA recruitment lead to insufficient TBK1 activation, markedly reduced phosphorylation of IRF3 and IRF7, and ultimately inhibition of IFN promoter activation and antiviral gene expression (6). Overall, RBM47 acts as an immune response “brake” in the early phase of viral infection by promoting MAVS lysosomal degradation and blocking signal complex assembly, preventing immune pathological damage caused by excessive IFN production. This regulatory mechanism is highly conserved in lower vertebrates and contributes to understanding the evolutionary characteristics of mammalian innate immune regulation (**Figure 4**). These findings are based on Level III evidence (zebrafish and cell-line models); independent mammalian validation for the MAVS degradation mechanism is lacking.

## 3 Clinical translation of RBM47

It is essential to recognize that all RBM47-associated biomarker or therapeutic claims discussed in the following sections remain at the preclinical, retrospective-correlative, or speculative stage. No prospective clinical trial, multi-center biomarker validation, or interventional pharmacodynamic study has been completed. We therefore distinguish three categories of translational evidence:Retrospective correlative associations: RBM47 expression correlates with TNM stage, survival, or immune marker expression in tissue cohorts. These observations generate hypotheses but do not establish clinical utility; Preclinical proof-of-concept: RBM47 overexpression or knockdown alters tumor growth, metastasis, or drug sensitivity in cell lines and xenografts. These findings provide mechanistic rationale but require extensive validation before clinical application; Speculative inferences: Correlations with PD-1, PD-L1, TIM-3, or NK-cell infiltration interpreted as immunotherapy response prediction remain hypothetical until tested in immune-competent *in vivo* models or treated patient cohorts.

### 3.1 Biomarker applications

RBM47 expression levels, pathway activity, and upstream epigenetic modification characteristics can cover the full clinical cycle of disease and serve as potential markers for diagnostic subtyping, prognostic stratification, and treatment response prediction in multiple tumor types and neurological diseases. However, all such applications remain at the retrospective or preclinical stage.

#### 3.1.1 Diagnosis and molecular subtyping

Expression differences in RBM47 enable precise identification of multiple diseases in preclinical models. In glioma, multi-cohort bioinformatic studies show that RBM47 is specifically highly expressed in the mesenchymal subtype of glioblastoma (GBM) (Chinese Glioma Genome Atlas [CGGA]/Repository for Molecular Brain Neoplasia Data [REMBRANDT]/The Cancer Genome Atlas [TCGA] cohorts, P < 0.0001) and can be used for auxiliary diagnosis of this highly aggressive molecular subtype in retrospective analyses (12, 73). In PTC, analysis based on the TCGA database and validation in 100 clinical paired samples show that RBM47 is markedly downregulated in PTC tissue (P < 0.01), with both mRNA and protein levels markedly lower than in adjacent normal tissue. Low expression correlates with larger tumor volume and higher tumor, node, metastasis (TNM) stage. Immunohistochemistry (IHC) shows a positive expression rate of only 43.3%, far lower than the 65.0% in adjacent normal tissue (P = 0.017), indicating its utility as an auxiliary diagnostic marker for preoperative biopsy differentiation of benign vs. malignant lesions in retrospective cohorts (10).

In NSCLC, existing clinical studies show heterogeneity in RBM47 expression trends: most studies report that low RBM47 expression correlates with advanced TNM stage, tumor emboli, and pleural invasion (31); database mining analysis that RBM47 expression is markedly higher in lung adenocarcinoma than in lung squamous cell carcinoma (P < 0.001), providing an auxiliary reference index for pathological subtyping in retrospective analyses (54). In CRC, a meta-analysis included 12 independent cohorts with 584 patients; 10 cohorts validated that RBM47 expression is markedly lower in CRC tissue than in adjacent tissue, with a fold change of 0.40 in the TCGA colon adenocarcinoma (COAD) cohort (P = 7.14 × 10^-^²¹), confirming its potential as an auxiliary diagnostic marker for CRC in retrospective database mining (13). In addition, preclinical studies of POCD show that RBM47 protein levels in the hippocampus of anesthetized/surgical mice are markedly elevated (P < 0.01) and correlate positively with cognitive impairment severity. Combined detection of its upstream transcription factor RUNX1 (Pearson correlation coefficient r = 0.88) can identify high-risk POCD populations before surgery in animal models (9). No human POCD biomarker validation has been reported.

#### 3.1.2 Prognostic assessment

The prognostic value of RBM47 shows clear disease specificity: in most tumors it exerts tumor-suppressive function, and low expression indicates poor prognosis in retrospective cohorts; in a minority of tumors it exerts oncogenic function, and high expression indicates poor prognosis in retrospective cohorts.

Low RBM47 expression correlates with poor prognosis in multiple solid tumors. Multivariate Cox analysis from a single retrospective cohort shows that low RBM47 expression is an independent poor prognostic factor for OS (HR = 2.716, 95% CI 1.598-4.725, P = 0.038) and relapse-free survival (RFS) (HR = 2.716, 95% CI 1.678-3.625, P = 0.042) in NSCLC patients, although the applicability of this correlation in specific populations requires further validation (31). In lung adenocarcinoma, low RBM47 expression or loss correlates with persistent Nrf2 pathway activation and also indicates poor prognosis in preclinical models (32). Based on the TCGA kidney renal clear cell carcinoma (KIRC) cohort and validation in 40 clinical paired samples, RBM47 expression in RCC correlates negatively with tumor grade, and high expression is an independent favorable predictor of OS (HR = 0.41, P = 4.4 × 10^-8^) (2). In PTC, low RBM47 expression correlates with larger primary tumor volume (P = 0.021) and higher TNM stage (stage III-IV, P = 0.006), indicating an invasive phenotype and poor prognosis in retrospective analyses (10). In BC, RBM47 loss correlates with enhanced metastatic capacity and can be used to identify high-risk metastatic patient subgroups in preclinical models (3). In CRC, multi-cohort studies show that in the TCGA COAD cohort, patients with low RBM47 expression have markedly shortened OS (P = 0.0071) and RFS (P = 0.0088); pooled analysis of 2173 patients shows that the low-expression group has a markedly elevated recurrence risk (log-rank P = 5.31 × 10^-5^). In CRC consensus molecular subtypes (CMS), the mesenchymal CMS4 subtype with the worst prognosis has the lowest RBM47 expression (z-score ≈ −1.5). Its promoter CpG methylation level correlates negatively with protein expression and positively with tumor stage; the methylation rate in patients with liver metastasis reaches 93%, making it a specific marker for CRC metastasis prediction in retrospective analyses (13).

RBM47 high expression correlates with poor prognosis mainly in PC and glioma. Pan-cancer analysis shows that RBM47 is markedly upregulated in PC tissue (|Log2FC| > 1, P < 0.05) and correlates positively with primary tumor T stage, indicating stronger local invasiveness in retrospective cohorts (15). In glioma, RBM47 expression correlates positively with WHO grade, IDH-wildtype, 1p/19q non-co-deletion, and unmethylated O-6-methylguanine-DNA methyltransferase (MGMT) promoter—molecular features associated with poor prognosis—and is an independent risk factor for shortened OS in retrospective bioinformatic cohorts (73). Multi-cohort analysis further shows that high RBM47 expression is an independent poor prognostic factor for OS (CGGA cohort HR = 1.047, P = 0.013; TCGA cohort HR = 1.334, P < 0.001). A nomogram prediction model constructed by combining WHO grade, IDH mutation status, and other clinical features achieves a concordance index (C-index) of 0.863 and can predict 1–10 year survival probability in retrospective analyses (12, 72). No prospective validation of these prognostic models has been conducted.

#### 3.1.3 Treatment response monitoring

RBM47 expression levels and pathway activity have been hypothesized to predict response rates to multiple treatment regimens in preclinical models, but these hypotheses remain untested in clinical cohorts. For solid tumors: in lung adenocarcinoma, the integrity of the RBM47-KEAP1-Nrf2 axis correlates with sensitivity to oxidative stress-inducing therapies (e.g., radiotherapy, cisplatin, paclitaxel) in cell lines (32); in RCC, patients with high RBM47 expression show an approximately 50% reduction in sunitinib half maximal inhibitory concentration (IC50) in cell-based assays, indicating higher sensitivity to targeted therapy in preclinical models (2); in NSCLC, high RBM47 expression reduces PD-L1 mRNA stability in cell lines, raising a hypothesis that RBM47 status might influence immune checkpoint blockade efficacy, but this has not been validated in any clinical cohort (42); in glioma, high RBM47 expression correlates highly with TIM-3 (r = 0.799-0.844) and PD-1 (r = 0.590) expression in retrospective transcriptomes, suggesting a hypothetical probability of benefit from TIM-3/PD-1 combined immunotherapy that remains entirely speculative (73); in PC, high RBM47 expression correlates negatively with NK cell infiltration in retrospective cohorts, indicating an “immune-cold” tumor phenotype, but whether this predicts poor response to immune checkpoint inhibitors has not been tested (15). For non-neoplastic diseases: in POCD, RBM47-cGAS-STING pathway activity, downstream inflammatory factors (TNF-α, IL-6, IFN-β), and ferroptosis indicators (malondialdehyde [MDA], iron content) can be used to evaluate the effect of anti-inflammatory therapy in animal models (9); in viral infectious diseases, dynamic RBM47 expression can evaluate host immune status and viral replication levels in zebrafish models, but this application currently lacks clinical research support (6).

### 3.2 Therapeutic target development

Based on the bidirectional functional characteristics of RBM47 in different diseases, current targeted intervention strategies are divided into two categories: expression activation (for scenarios where RBM47 exerts tumor-suppressive function but is downregulated) and expression or functional inhibition (for scenarios where RBM47 exerts oncogenic function and is upregulated). All such strategies remain at the preclinical proof-of-concept stage.

#### 3.2.1 Monotherapy development

For tumor-suppressive scenarios with low RBM47 expression (activation required): (1) Epigenetic modulators: the DNA methyltransferase inhibitor 5-azacytidine (5-Aza) combined with the histone deacetylase (HDAC) inhibitor trichostatin A (TSA) restores expression of methylated-silenced RBM47 mRNA in CRC cells and effectively inhibits tumor cell invasion *in vitro* (13). HDAC inhibitors or histone acetyltransferase activators can upregulate H3K27ac levels at the RBM47 promoter to restore RBM47 expression in RCC cell lines (2). (2) miRNA inhibitors: anti-miR-181c/d-5p relieves post-transcriptional suppression of RBM47 by miRNA in CRC and upregulates RBM47 expression approximately 3-fold in cell lines, inhibiting tumor proliferation and invasion (14). (3) Natural products: Platycodin D (saponin from *Platycodi radix*) downregulates miR-181c/d-5p expression and upregulates RBM47, inhibiting CRC proliferation and invasion in cell lines; *in vivo* experiments show suppression of subcutaneous tumor growth in xenograft models (14). (4) Gene therapy: lentivirus-mediated RBM47 overexpression reduces tumor volume and weight by approximately 60% in a PTC nude mouse xenograft model (10) and also inhibits xenograft growth in RCC (2).

For oncogenic scenarios with high RBM47 expression (inhibition required): (1) Small molecule/peptide inhibitors: specific inhibitors could be developed to block binding between the RBM47 RRM2 domain and the *cGAS* mRNA 3′-UTR (the “UUUCUUUGUUUG” sequence), reducing *cGAS* mRNA stability and inhibiting excessive cGAS-STING activation in POCD in preclinical models (9). This remains a theoretical design without experimental validation. (2) Natural products: the natural steroidal saponin dioscin crosses the blood-brain barrier, downregulates RBM47 expression through NF-κB pathway inhibition (protein level reduced by approximately 50%, P < 0.05), suppresses M2 macrophage polarization in glioma cell lines, and delays orthotopic tumor growth in xenograft models (12). (3) Upstream pathway inhibitors: RUNX1 is an upstream transcriptional activator of RBM47; use of RUNX1 inhibitors may indirectly downregulate RBM47 expression and provide an upstream intervention for POCD in preclinical models (9). (4) Gene therapy: microglia-specific adeno-associated virus serotype 9 (AAV9)-short hairpin RNA (shRNA) against RBM47 can precisely silence central RBM47 and achieve targeted intervention for POCD in animal models (9).

#### 3.2.2 Combination therapy

Multiple combination regimens have shown concurrent effects in preclinical studies according to disease molecular mechanisms, but no combination pharmacodynamic data in patients exist:

##### 3.2.2.1 Solid tumor field

In lung adenocarcinoma, RBM47 activators have been hypothesized to be combined with oxidative stress-inducing agents such as radiotherapy, cisplatin, or paclitaxel, or with Nrf2 inhibitors or KEAP1 activators, to enhance tumor cell sensitivity to oxidative stress in cell lines (32). In NSCLC, RBM47 has been shown to enhance T cell-mediated antitumor activity in preclinical models (42); however, the hypothesis that RBM47 activators combined with PD-1/PD-L1 inhibitors would relieve T cell suppression remains speculative and has not been tested experimentally. In RCC, RBM47 activators combined with sunitinib reduce tumor weight more than sunitinib alone in xenograft models and may overcome targeted drug resistance (2). In PC, RBM47 inhibitors combined with PD-1/PD-L1 inhibitors have been proposed to downregulate PD-L1 expression and reverse immune suppression; combined with NK cell adoptive therapy, NK cell cytotoxicity increases from approximately 15% to 45% in co-culture experiments (15). In glioma, RBM47 inhibitors combined with TIM-3/PD-1 inhibitors have been proposed to relieve immune suppression in preclinical models (73); combined with temozolomide or cisplatin, they may reverse EMT-mediated chemoresistance in cell lines. In CRC animal models, RBM47 upregulation combined with PI3K/AKT inhibitors or chemotherapeutic drugs shows a concurrent anti-tumor trend (62, 63).

##### 3.2.2.2 Non-neoplastic field

In POCD animal models, AAV9-shRBM47 combined with the cGAS inhibitor RU.521 concurrently improves cognitive function, inhibits neuroinflammation and ferroptosis, with stronger effects than either monotherapy in mice (9). In viral infection treatment, controllable RBM47 inhibitors could serve as antiviral adjuvant therapy in theoretical models, but the degree and timing of inhibition must be strictly controlled to avoid autoimmune pathological damage (6).

Currently, all RBM47-targeted therapies are in the preclinical concept-validation stage. Natural products such as dioscin and Platycodin D have validated safety and efficacy in multiple animal models, and no specific RBM47-targeted drug has entered clinical trials (**Table 4**).

**Table 4:** Clinical translational strategies: biomarker applications and targeted interventions.

Translational direction	Disease context	Application type	Molecular / biological basis	Intervention / diagnostic strategy	Evidence stage	Refs
Biomarker	Glioma	Prognostic / Molecular subtyping	Mesenchymal subtype-specific high expression; IDH-wildtype correlation; TIM-3 / PD-1 co-expression	Composite panel (RBM47 + TIM-3 / PD-1) for prognostic stratification and immunotherapy benefit prediction	Level II/III: Retrospective bioinformatic cohorts (CGGA, TCGA, REMBRANDT). Hypothetical inference for immunotherapy prediction. No prospective validation.	(12, 73)
	Papillary thyroid carcinoma	Diagnostic / Prognostic	Significantly downregulated in tumor tissues; inversely correlates with tumor size and TNM stage	IHC / qPCR profiling for benign vs. malignant differentiation and invasion risk assessment	Level II: Single-center retrospective cohort validation (n=100). No multi-center prospective trial.	(10)
	Colorectal cancer	Diagnostic / Prognostic / Predictive	CMS4 subtype association; promoter methylation correlates with liver metastasis	Combined RBM47 expression and methylation status testing for metastasis / recurrence prediction	Level II: TCGA & multi-cohort retrospective mining. Level III: 5-Aza/TSA only *in vitro*. No prospective liquid-biopsy validation.	(13)
	Renal cell carcinoma	Prognostic / Companion diagnostic	High expression predicts favorable OS and sunitinib sensitivity (IC_50_ reduced by ~50%)	Expression profiling to guide targeted therapy selection	Level II: Single-center retrospective cohort (n=40). Cell-based IC_50_ assay. No prospective companion diagnostic trial.	(2)
	Postoperative cognitive dysfunction	Risk prediction	Hippocampal upregulation; highly co-expressed with RUNX1 (r = 0.88)	Peripheral blood / CSF dual detection of RBM47 and RUNX1	Level III: Preclinical (animal model only). No human biomarker validation.	(9)
Therapeutic target	Colorectal cancer / Breast cancer / NSCLC / RCC / Papillary thyroid carcinoma	Epigenetic reactivation / Overexpression	Silenced by promoter hypermethylation or miR-181c/d-5p targeting	Demethylating agents (5-Aza + TSA); anti-miR-181c/d-5p; Platycodin D; lentiviral overexpression	Level III: Preclinical (cell / animal models only). No Phase I clinical trial.	(2, 10, 13, 14, 62)
	Glioma / Pancreatic cancer	Targeted inhibition / Silencing	High expression drives malignant progression, M2 polarization, and immune evasion	Dioscin; RUNX1 inhibitors; specific shRNA / siRNA-mediated silencing	Level III: Preclinical (cell / animal models only). No specific small-molecule inhibitor in clinical development.	(12, 15)
	Postoperative cognitive dysfunction	Functional inhibition	Activates cGAS-STING pathway, ultimately leading to neuronal ferroptosis	AAV9-mediated shRBM47 delivery; cGAS specific inhibitors (e.g., RU.521)	Level III: Preclinical (animal model only). No human interventional data.	(9)
Combination therapy	Pan-cancer	Immune + targeted combination	RBM47 directly modulates PD-L1 levels, NK cell cytotoxicity, and M2 macrophage polarization	RBM47 agonists / inhibitors combined with ICIs (anti-PD-1 / PD-L1), sunitinib, or chemotherapy	Level IV: Hypothetical inference / concurrent administration in cell lines. No combination pharmacodynamic data in patients. No clinical trial.	(2, 15, 32, 42)

### 3.3 Evidence stratification and clinical readiness

Current evidence for RBM47 clinical translation is characterized by a uniform limitation in study design. All clinical biomarker data derive from retrospective analyses: single-center cohorts for PTC (n = 100) (10), RCC (n = 40) (2), and NSCLC (n = 136) (31), alongside public database mining (TCGA, CGGA, GEO, REMBRANDT) that carries inherent selection biases and batch effects. No multi-center prospective validation has been conducted. All therapeutic strategies—including epigenetic modulators, miRNA inhibitors, natural products, and gene therapy—remain at the preclinical proof-of-concept stage in cell and animal models. As of this writing, no RBM47-targeted agent has entered phase I clinical trials.

### 3.4 Core challenges in clinical translation

Three main challenges currently constrain the clinical translation of RBM47. (1) Off-target risk from functional heterogeneity: RBM47 has bidirectional functions in different diseases (e.g., oncogenic in PC but tumor-suppressive in most solid tumors). Systemic administration can cause functional damage in non-target organs. For example, RBM47 must be inhibited in PC but activated in CRC and RCC; in a patient with both tumors, the choice is contradictory. In addition, RBM47 must be inhibited in microglia for POCD, but systemic inhibition may increase susceptibility to viral infection (based on the function of RBM47 in stabilizing IFNAR1 to enhance antiviral immunity in mammalian cells) or induce autoimmunity (based on the function of RBM47 in promoting IL-10 in B cells). Tissue-specific delivery systems are required to ensure drug safety, but such systems remain underdeveloped. (2) Poor druggability: RBM47 functions depend on RRM domain-RNA interactions rather than a typical enzymatic active pocket. Traditional high-throughput screening methods are poorly suited to discovering small molecules that target protein-RNA interactions. No specific small molecule targeting RBM47 has entered clinical development. Although RBM39 can be targeted by molecular glue degraders, whether RBM47 possesses similar structural features is unclear. (3) Difficulty in precise regulation: clinical application requires strict control of RBM47 expression or activity levels to avoid side effects from excessive activation or inhibition (e.g., over-inhibition inducing autoimmunity in antiviral therapy). Technologies for spatiotemporally controllable regulation are currently lacking.

Future research must focus on resolving the disease-specific target mRNA networks of RBM47, developing highly specific small-molecule modulators (e.g., PROTACs, molecular glues), and exploring patient stratification treatment strategies based on RBM47 expression levels to advance clinical translation.

## 4 Summary and prospects

RBM47 is a highly conserved multifunctional RNA-binding protein. Through its three RRM domains, it mediates key post-transcriptional events, including C-to-U RNA editing, alternative splicing, and mRNA stability regulation. Under physiological conditions, RBM47 is essential for vertebrate embryonic development, stem cell differentiation, and immune homeostasis maintenance. In disease contexts, RBM47 exhibits a highly context-dependent, dual regulatory role: in BC, CRC, NSCLC, RCC, and PTC, preclinical evidence suggests that it exerts a tumor-suppressive function by inhibiting the Wnt/β-catenin, PI3K/AKT, and Nrf2 pathways, and activating p53 and autophagy signaling. Conversely, in glioma and PC, cell and animal models indicate that it drives malignant progression by stabilizing oncogenic target mRNAs (e.g., TRAF6, PDIA6), promoting M2 macrophage polarization, and inducing immune evasion. Additionally, dysregulated RBM47 expression contributes to non-neoplastic conditions, including POCD (via cGAS-STING-mediated neuronal ferroptosis in mouse models), IBD (conferring acute protection while promoting long-term carcinogenesis in conditional knockout models), and antiviral innate immunity (via MAVS lysosomal degradation in zebrafish).

The expression level, promoter methylation status, and downstream signaling networks of RBM47 show preclinical promise as potential biomarkers for diagnostic subtyping, prognostic stratification, and treatment response prediction across multiple diseases, pending prospective validation in independent cohorts and standardized assay development. Currently, preclinical intervention strategies targeting RBM47 (such as epigenetic activation, miRNA inhibition, natural products, gene therapy, and combination therapies) have shown preliminary efficacy in cell and animal models. However, three major bottlenecks hinder its clinical translation: (1) functional pleiotropy poses an off-target risk for systemic interventions; (2) as an RNA-binding protein lacking a classical small-molecule binding pocket, its druggability is substantially limited; (3) technologies for spatiotemporally controlled and tissue-specific delivery remain underdeveloped.

Future research directions should include: (1) Mechanism level: mapping the transcriptome-wide target spectrum of RBM47 across different cell types and elucidating the molecular switches (e.g., cofactors, post-translational modifications) that dictate its functional switching. (2) Technology level: developing highly specific protein-RNA interaction inhibitors (such as PROTACs or molecular glue-based targeted degradation strategies) and tissue-specific delivery vectors (e.g., AAV serotype screening, lipid nanoparticle modifications). (3) Clinical level: conducting prospective, multi-center cohort studies to validate the utility of RBM47 as a liquid biopsy marker and exploring stratified therapeutic strategies based on RBM47 expression profiles.

In conclusion, RBM47 exemplifies the context-dependent “double-edged sword” nature of RNA-binding proteins in disease. However, it is critical to emphasize that all biomarker associations, therapeutic hypotheses, and combination strategies discussed in this review remain at the preclinical or retrospective-correlative stage. No RBM47-targeted agent has entered Phase I clinical trials. The functional pleiotropy of RBM47 across different tissues poses a substantial off-target risk for systemic interventions, and the lack of a classical enzymatic active pocket limits conventional small-molecule drug development. Future research must prioritize: (1) mapping disease-specific target mRNA networks to resolve the molecular switch governing functional duality; (2) developing highly specific protein-RNA interaction inhibitors with tissue-specific delivery vectors; and (3) conducting prospective, multi-center cohort studies to validate RBM47 as a biomarker before any clinical application can be considered.

## Glossary

5-Aza: 5-Azacytidine
AAV9: Adeno-associated virus serotype 9
A1CF: APOBEC1 complementation factor
AKT: Protein kinase B
Apob: Apolipoprotein B
APOBEC1: Apolipoprotein B mRNA editing enzyme catalytic subunit 1
AREG: Amphiregulin
ATF5: Activating transcription factor 5
CASP3: Caspase-3
CARD: Caspase activation and recruitment domain
CAC: Colitis-associated cancer
CCN1: Cellular communication network factor 1
CD133: Prominin-1
CD90: Thy-1 cell surface antigen
CGGA: Chinese Glioma Genome Atlas
C-index: Concordance index
CI: Confidence interval
CMS: Consensus molecular subtype
CRC: Colorectal cancer
CTTN: Cortactin
CUL3: Cullin 3
Cryo-EM: Cryo-electron microscopy
DSS: Dextran sulfate sodium
EGFR: Epidermal growth factor receptor
EMT: Epithelial-mesenchymal transition
ERK: Extracellular signal-regulated kinase
ESC: Embryonic stem cell
F-actin: Filamentous actin
FNDC5: Fibronectin type III domain containing 5
FOXA1: Forkhead box A1
FOXO3: Forkhead box O3
GADD45A: Growth arrest and DNA damage inducible alpha
GADD45B: Growth arrest and DNA damage inducible beta
GPX4: Glutathione peroxidase 4
GWAS: Genome-wide association study
HDAC: Histone deacetylase
HO-1: Heme oxygenase-1
HOXB-AS1: HOXB cluster antisense RNA 1
HR: Hazard ratio
H3K27ac: Histone H3 lysine 27 acetylation
IBD: Inflammatory bowel disease
IC50: Half maximal inhibitory concentration
ICI: Immune checkpoint inhibitor
IDH: Isocitrate dehydrogenase
IFN: Interferon
IFNAR1: Interferon alpha and beta receptor subunit 1
IFN-β: Interferon-beta
IHC: Immunohistochemistry
IL-1A: Interleukin-1 alpha
IL-1B: Interleukin-1 beta
IL-6: Interleukin-6
IL-10: Interleukin-10
IL-33: Interleukin-33
ILC2: Type 2 innate lymphoid cells
IRF3: Interferon regulatory factor 3
IRF7: Interferon regulatory factor 7
KEAP1: Kelch-like ECH-associated protein 1
lncRNA: Long non-coding RNA
Lgr5: Leucine-rich repeat-containing G protein-coupled receptor 5
Lrig1: Leucine-rich repeats and immunoglobulin-like domains 1
MAVS: Mitochondrial antiviral-signaling protein
MDA: Malondialdehyde
MDA5: Melanoma differentiation-associated protein 5
MEF2C: Myocyte enhancer factor 2C
MGMT: O-6-methylguanine-DNA methyltransferase
miRNA: MicroRNA
MITA: Mediator of IRF3 activation
mRNA: Messenger RNA
NMR: Nuclear magnetic resonance
NK: cell Natural killer cell
NQO1: NAD(P)H quinone oxidoreductase 1
NSCLC: Non-small cell lung cancer
Nrf2: Nuclear factor erythroid 2-related factor 2
NF-κB: Nuclear factor kappa B
OS: Overall survival
p21: Cyclin-dependent kinase inhibitor 1A
PC: Pancreatic cancer
PD-1: Programmed cell death protein 1
PD-L1: Programmed death-ligand 1
PDIA6: Protein disulfide isomerase family A member 6
PI3K: Phosphoinositide 3-kinase
poly I:C: Polyinosinic:polycytidylic acid
POCD: Postoperative cognitive dysfunction
PrE: Primitive endoderm
PROTAC: Proteolysis targeting chimera
PTC: Papillary thyroid carcinoma
PTEN: Phosphatase and tensin homolog
qPCR: Quantitative polymerase chain reaction
RBM: RNA-binding motif
RBM47: RNA-binding motif protein 47
RCC: Renal cell carcinoma
REMBRANDT: Repository for Molecular Brain Neoplasia Data
RFS: Relapse-free survival
RIG-I: Retinoic acid-inducible gene I
RLR: RIG-I-like receptor
ROS: Reactive oxygen species
RRM: RNA recognition motif
RUNX1: Runt-related transcription factor 1
shRNA: Short hairpin RNA
siRNA: Small interfering RNA
SNAI1: Zinc finger protein SNAI1 (SNAIL)
SNHG5: Small nucleolar RNA host gene 5
SNP: Single nucleotide polymorphism
SVCV: Spring viremia of carp virus
STAT3: Signal transducer and activator of transcription 3
TBK1: TANK-binding kinase 1
TCA: cycle Tricarboxylic acid cycle
TCGA: The Cancer Genome Atlas
TJP1: Tight junction protein 1
TME: Tumor microenvironment
TIM-3: T-cell immunoglobulin and mucin-domain containing-3
TNF-α: Tumor necrosis factor alpha
TRAF6: TNF receptor associated factor 6
TNM: Tumor, node, metastasis
TSA: Trichostatin A
USP21: Ubiquitin-specific peptidase 21
UTR: Untranslated region
VIG1: Virus-induced gene 1
WHO: World Health Organization
YBX3: Y-box binding protein 3
ZO-1: Zonula occludens-1
